# Efficacy of HIV interventions among factory workers in low- and middle-income countries: a systematic review

**DOI:** 10.1186/s12889-020-09333-w

**Published:** 2020-08-28

**Authors:** Dahui Chen, Ganfeng Luo, Xiaojun Meng, Zixin Wang, Bolin Cao, Tanwei Yuan, Yu Xie, Tian Hu, Yaqi Chen, Wujian Ke, Zhenyu Wang, Caijun Sun, Kai Deng, Yong Cai, Kechun Zhang, Huachun Zou

**Affiliations:** 1 School of Public Health (Shenzhen), Sun Yat-sen University, Shenzhen, Guangdong PR China; 2 Wuxi Center for Disease Control and Prevention, Wuxi, Jiangsu PR China; 3 Centre for Health Behaviours Research, JC School of Public Health and Primary Care, Faculty of Medicine, The Chinese University of Hong Kong, Hong Kong, PR China; 4 School of Media and Communication, Shenzhen University, Shenzhen, Guangdong PR China; 5 Longhua District Center for Disease Control and Prevention, Shenzhen, Guangdong PR China; 6 Dermatology Hospital, Southern Medical University, Guangzhou, Guangdong PR China; 7 School of Public Health, Sun Yat-sen University, Guangzhou, Guangdong PR China; 8 Zhongshan School of Medicine, Sun Yat-sen University, Guangzhou, Guangdong PR China; 9 Department of Community Health and Family Medicine, School of Public Health, Shanghai Jiao Tong University, Shanghai, PR China; 10 Kirby Institute, University of New South Wales, Sydney, Australia

**Keywords:** HIV, Factory workers, HIV-related risk behaviors, Interventions, Low- and middle-income countries

## Abstract

**Background:**

Factory workers in low- and middle-income countries (LMICs) are vulnerable to HIV transmission. Interventions are needed to prevent HIV in this population. We systematically reviewed published literature on the efficacy of various HIV interventions in reducing stigma, risk behaviors and HIV transmission among factory workers.

**Methods:**

A systematic review was performed using predefined inclusion and exclusion criteria. Four databases (PubMed, PsycINFO, Scopus and EMBASE) were searched for relevant publications between January 1, 1990 and December 31, 2018. Two independent reviewers assessed the methodological quality of studies.

**Results:**

Thirteen articles were included, with 2 randomized controlled trials and 11 cohort studies. Five interventions and their combinations were summarized. Educational intervention increased condom use and reduced the use of recreational drugs and alcohol before sex. Community intervention that proactively provide HIV counselling and testing (HCT) services could increase the detection rate of HIV and other sexually transmitted diseases (STDs). Lottery intervention increased HCT uptake and decreased HIV public stigma. Education combined with community intervention reduced the proportion of workers with casual sex and enhanced HIV knowledge. Peer education combined with community intervention increased the proportion of workers who were willing to take their partners to HCT. Policy intervention combined with peer education enhanced HIV knowledge, perceived condom accessibility and condom use with regular partners.

**Conclusions:**

Various interventions improved HIV knowledge, decreased HIV stigma and reduced HIV-related risk behaviors among factory workers in LMICs. The combination of multiple interventions tended to achieve better efficacy than a single intervention. Persistent combination interventions are essential to address HIV in this population.

## Background

Human immunodeficiency virus (HIV) infection is a major public health challenge and a major disease burden in low- and middle-income countries (LMICs) [1]. According to the United Nations Program on HIV/AIDS (UNAIDS) in 2016, about 36.7 million people in the world were living with HIV/AIDS, 95% of whom were from LMICs [2]. Existing studies have shown that demographic characteristics which were associated with increased risk of HIV infection included low level of education [3], unprotected sex [4], and labor-intensive enterprise workers including factory workers [5, 6].

Factory workers are especially vulnerable to HIV. A cohort study in Ethiopia showed that 8.5% of workers in factories were infected with HIV, with an incidence of 0.4 per 100 person-years [7]. According to study published in 2019, Lesotho national textile factory workers had higher HIV prevalence than other adults in the country (42.7% vs 25%, *P* < 0.05) [6]. A cross-sectional study in 2016 in China also found that factory workers were more likely to be HIV positive than students in the screening of blood donors (0.1% vs 0.03%, *P* < 0.01) [8]. Various factors influence the susceptibility of factory workers to HIV. The first reason is that this group are sexually active and had insufficient education. According to the literature [7, 9], more than 63% of factory workers were under the age of 35 and more than 44% had less than junior high school education. The second reason is the high prevalence of high-risk sexual behaviors. Available data showed that over 40% of factory workers had two or more sex partners in the past 12 months [7, 9, 10]. Approximately 20% of factory workers had sex with non-regular partners in the last 6 months, with whom 66.3% used alcohol before sex [10]. Studies showed that drinking alcohol before sex increased the risk of HIV infection [11, 12]. Finally, just over 40% of this population would like to accept HIV counselling and testing (HCT) when this service is available [13]. HIV-related stigma may prevent them from accepting HCT.

In LMICs, health education and behavioral interventions have been playing an essential role in the control of HIV. A randomized controlled trial (RCT) in Lithuania showed that health education has a significant impact on improving HIV knowledge and reducing HIV infection risk behaviors, but has little effect on attitude change [14]. However, another intervention-peer education, not only improved HIV knowledge and changed HIV attitude, but improved HCT uptake [15, 16]. Recently, an RCT in Zimbabwe demonstrated that providing financial incentive could also significantly improve HCT uptake [17]. These useful interventions, including educational intervention, peer education, and financial incentives, could produce beneficial outcomes among factory workers [18–20]. However, to our knowledge, there was no published review that summarizes and compares the efficacy of these interventions among factory workers. We performed a systematic review to evaluate various interventions reported in published articles involving factory workers in LMICs in reducing HIV infection, changing HIV risk behaviors and attitudes, and decreasing HIV stigma.

## Methods

This systematic review followed the guidelines set forth in the 2010 ‘Preferred Reporting Items for Systematic Reviews and Meta-Analyses (PRISMA)’ [21]. In addition, we developed a protocol, but it was not registered or published (see Additional file 1).

### Search strategy

In this paper, studies about factories (also known as manufacturing plants) or people working in factories were included. The Mesh terms “workplace/industry/acquired immunodeficiency syndrome” and the key word “worker” were combined using the Boolean operator and with the following key words: (enterprise, firm, company, workshop, floor shop, machine shop, mill, factory, manufactory) and (worker, workman, workingman, employee). Key words in parentheses were connected to operators. The search strategy was implemented in the databases PubMed, PsycINFO, Scopus and EMBASE using a date range of January 1, 1990 through December 31, 2018. Grey literature online (e.g., AIDS Conference, International AIDS Society Conference), was also searched, but no relevant articles were found. Details of the search strategies are presented in Additional file 2: Table S1.

Two authors of this study (DC and GL) each independently searched for relevant articles. Titles, abstracts, full texts and reference lists of all identified reports were reviewed in duplicate by the two authors, and extracted articles were double-checked. Disagreements were resolved by discussion among the three authors (DC, GL and HZ). Reference lists from related main studies and review articles were also checked for additional relevant reports.

### Eligibility criteria

The inclusion criteria were: (1) The scope was formulated using the population, intervention, comparison, outcomes, and study design (PICOS) [22, 23] format, which are listed in Table 1; (2) Studies were conducted in LMICs according to the World Bank (the World Bank classifies countries by income. Countries can be categorized into LMICs and High-Income Countries (HICs). The LMIC status of a country where an included study was conducted was judged according to the World Bank classification of country by income in 2017, the most up-to-date data when we conducted our review) [24]; (3) Studies report on a specific intervention time span; (4) Articles were written in English. In order to ensure that we were not overlooking relevant studies, we had no restrictions on intervention methods.

**Table 1:** Population, intervention, comparison, outcome, and study design (PICOS) criteria for study inclusion

Criteria	Definition
Population	Factory workers
Intervention	Interventions aimed at reducing HIV incidence, stigma, risk behaviors, changing HIV attitude and increasing HIV/AIDS knowledge and HIV counseling and testing (HCT) uptake
Comparison	Comparison between pre- and post-intervention periods or between intervention and control groups
Outcome	HIV incidence, stigma, knowledge, attitude, risk behaviors, uptake of HCT
Study Design	Pre- and post-intervention study design, randomized controlled trial, and quasi-experiment

Studies were excluded based on the following criteria: (1) Participants were not factory workers; (2) No intervention; (3) Article published before 1990; (4) Studies were observational, and did not describe an intervention’s efficacy on reducing HIV infection, changing HIV risk behaviors and attitudes, and decreasing HIV stigma (e.g., a cross-sectional study); (5) Systematic review, literature review, case series; (6) Article published in languages other than English.

### Data extraction and statistical analysis

The following data were extracted from publications: year of publication, first author, country in which the study took place, study design, sample size, length of follow-up, intervention method, and intervention outcomes. In addition, for studies that did not provide a *χ*^2^ value, we calculated the *χ*^2^ value using R version 3.6.0, including *P* value, if the necessary figures were provided in the paper.

### Quality assessment of included studies

In order to assess the quality of the included articles, we used the Quality Assessment Tool for Quantitative studies from the Effective Public Health Practice Project (EPHPP) [25, 26]. This tool has been widely used in literature to evaluate randomized control trials of HIV research [27, 28], and was recommended by the Cochrane Library in the area of Health Promotion and Public Health [29, 30]. Quality assessment included six components: selection bias, study design, confounders, blinding methods, data collection method, and withdrawals and drop-outs. The scores of each component are based on the documents [25, 26]. A study will receive a “strong” overall rating when none of the individual components has been rated as “weak”. If a study cannot get more than one “weak” score on any single component, it will receive a “medium” overall rating. A study of at least two “weak” ratings for individual components will be given a “weak” overall rating. Two authors (DC and GL) independently conducted quality assessment of included articles. If the quality evaluation results were different, the authors (DC and GL) would recheck the original article, and finally disagreements were resolved by discussion with the corresponding author (HZ)”.

## Results

### Overview of included studies

**Fig. 1:**
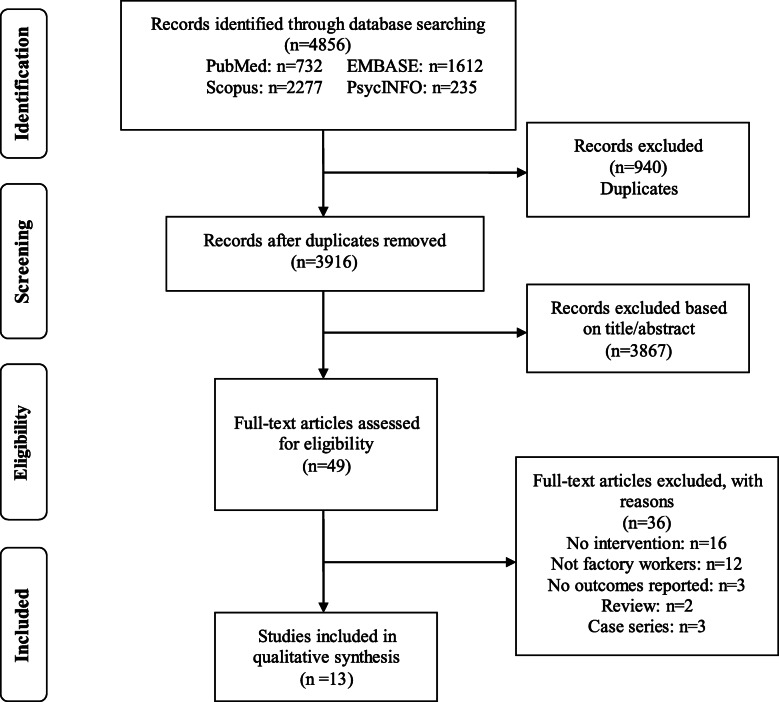
Flow chat of systematic review

Figure 1 shows the procedure of study inclusion. We identified 4856 articles using the specified search criteria (PubMed: *n* = 732; EMBASE: *n* = 1612; Scopus: *n* = 2277; PsycINFO: *n* = 235). 940 duplicated papers were removed. Based on the inclusion criteria, 3867 papers were excluded. 49 papers remained for full text review, and 13 papers met inclusion criteria.

Table 2 provides details regarding the 13 studies included in the review. Studies included in our review were published between 1996 and 2018. All countries included in our review either remained as low/mid-income or progressed from low to mid-income, and none of these countries progressed to a higher income status, according to the World Bank [24]. Eight took place in Africa (three in Zimbabwe [19, 31, 32], two in South Africa [20, 33], two in Ethiopia [34, 35] and one in Tanzania [36]), while the remaining five were performed in Asia (three in Thailand [18, 37, 38] and two in China [39, 40]). Two were RCTs [31, 37] and eleven were cohort studies [18–20, 32–36, 38–40]. All studies reported a statistically significant effect on one or more outcomes, which were reported as follows: eight reported HIV risk behaviors [18, 34–40], four reported HIV/AIDS knowledge [18, 37, 38, 40], four reported HCT uptake [19, 20, 32, 33], one reported HIV public stigma [20], one reported HIV/AIDS attitude [18] and one reported HIV incidence [31]. Risk behaviors included presence of multiple sex partners, commercial sex, recreational drugs or alcohol use before sex, and condom use, etc. According to the UNAIDS [41], the definition of HIV public stigma was a process of devaluation of people either living with or associated with HIV infection, such as, those who blamed foreigners/migrant workers/prostitutes for spreading HIV/AIDS.

**Table 2:** Characteristics of 13 studies of HIV interventions among factory workers in LMICs

	First author	Published year	Study design	Region	Length of follow-up	Intervention type
	Kuchaisit C	1996	RCT	Thailand	12 months	Education
**Sample size (before/after)**	Intervention group: 153 workers/133 workers; Control group: 148 workers/127 workers					
**Intervention**	**1.** Health workers organized a 20-min presentation of HIV every two weeks, using slides, brochures, and two-way presentations. **2.** Communication regarding AIDS, correct use of condoms, and posters exhibition.					
**Outcome**	**1.** Contact with extra-partner in the past 12 months (intervention group from 16 to 5%, *P* = 0.021). **2.** Unprotected sex in the past 12 months (slight decrease, 3% in control, 6% in education group, *P* < 0.05). **3.** Knowledge of HIV were found significantly higher in the education group compared to the control group (*P* < 0.05).					
	Sakondhavat C	1998	Cohort	Thailand	12 months	Education
**Sample size (before/after)**	305 workers /288 workers					
**Intervention**	**1.** Health workers provided cartoons, posters, radios, television, lectures and brochures about HIV/AIDS. **2.** Over the past 12 months, three in-depth interviews were conducted with workers to understand changes in their HIV knowledge, attitudes and high-risk behavior.					
**Outcome**	**1.** In the past 12 months, the proportion of workers who drank alcohol before sex decreased from 17 to 6.3% (*P* < 0.01) and took recreational drugs decreased from 2.6 to 0.7% (*P* < 0.01). **2.** The proportion of workers who did not use condoms during extramarital or premarital sex in the last 12 months decreased from 6.9 to 3.8% (*P* < 0.01) **3.** HIV/AIDS prevention and transmission knowledge improved (*P* < 0.05). **4.** HIV negative attitude decreased from 46.6 to 30.6% (*P* < 0.001).					
	Bassett M	1998	RCT	Zimbabwe	6 months	Combination of peer education and community intervention
**Sample size (before/after)**	Intervention group: 20 factories (2219 workers /1731 workers) Control group: 20 factories (NR)					
**Intervention**	**1.** Providing HCT services and Sexually Transmitted Disease (STD) treatment. **2.** One peer educator trained 100 workers, maintained a continuous supply of free condoms at worksites, and organized at least one drama and two presentations by persons living with HIV/AIDS, including one man and one woman. **3.** Peer educators led discussions, showed videos and slide shows.					
**Outcome**	HIV infection rates in the intervention group were 40% lower than in the control group (1.51 vs. 2.52 per 100 persons-years, *P* < 0.05).					
	Machekano R	1998	Cohort	Zimbabwe	28 months	Community intervention
**Sample size (before/after)**	2414 workers /2060 workers					
**Intervention**	Provide HCT (during recruitment and follow-up period), including individual risk assessment, discussion of HIV risk factors and modes of transmission, the meaning of test results and preventing HIV, and availability of treatment and support.					
**Outcome**	**1.** Workers at high risk behaviors were more likely to go to HCT, and proactive provision of HCT could increase the detection rate of HIV (relative risk (RR): 1.87, 95% confidence interval (CI): 1.01 to 3.61) and STD (RR: 3.47, 95%CI: 2.51 to 4.89). **2.** After 28 months, among men who went to HCT, a non-significant 40% decrease in HIV seroconversion (4.82 vs. 3.04 per 100 person-years, *P* = 0.18) and 30% increase in STDs incidence (10.84 vs. 14.79 per 100 person-years, *P* = 0.11) was observed compared to before. **3.** In the second follow-up, HIV seroconversion was higher among subjects who obtained their test results at the first follow-up compared to those who did not (19.5% vs. 16.7%, respectively, *P* = 0.01)					
	Qian X	2007	Cohort	China	6 months	Education
**Sample size (before/after)**	Intervention group: 340 workers /258 workers Control group: 257 workers /168 workers					
**Intervention**	**1.** Health workers disseminated knowledge and information about contraceptive and condom use to factory workers. **2.** Lectures given by experts, content about STD prevention. **3.** Distributing free condoms and contraceptives and providing HIV/STD counselling service.					
**Outcome**	**1.** Contraception use has increased from 70 to 93% in the past three months (*P* < 0.05). **2.** Condom use has increased from 41 to 70% in the past three months (*P* < 0.05).					
	Zhu C	2014	Cohort	China	9 months	Combination of education and community intervention
**Sample size (before/after)**	Intervention group: (2980 workers /1425 workers) Control group: (1060 workers /2139 workers)					
**Intervention**	**1.** Health workers provided sexual health education (Knowledge about healthy sexual activities; STD and HIV/AIDS knowledge, effects, prevention, symptoms; appropriate ways to obtain health care for STD, HIV/AIDS). **2.** Providing HCT services and promoting mental and physical health, such as mental health, reasonable diet and exercise. Disease and injury prevention, such as influenza or workplace injury prevention.					
**Outcome**	**1.** The rate of change of the intervention group who gave correct answers to the HIV/AIDS knowledge was significantly higher than that of control group (3.5% vs 1.1%, *P* < 0.05). **2.** In the intervention group, the proportion of workers who knew where provided free educational counselling was improved (3.5 to 6.7%, *P* < 0.001). **3.**The proportion of workers who had premarital sexual behaviors in intervention group was lower than control group (10.9% vs 31.3%, *P* < 0.001).					
	Ng’weshemi J.	1996	Cohort	Tanzania	22 months	Combination of education and community intervention
**Sample size (before/after)**	1433 workers /752 workers					
**Intervention**	**1.** Health workers provided free and effective treatment of STD and testing HIV antibody every 5.5 months. **2.** Health workers provided free condoms and HCT services in study clinic and factory. **3.** Health workers provided health education activities, including information about HIV/AIDS, drama performance.					
**Outcome**	**1.** Sexual partners: At a total of four follow-up at 22 months, the proportion of workers with sexual partners changed to: having one sexual partner (57.6, 68.4, 67.3, 70.2, and 72.9%, *P* < 0.001), having two sexual partners (17.6, 14.0, 12.2, 10.6, and 10.2%, *P* < 0.001), having three or more sexual partners (4.7, 4.1, 3.1, 2.3, and 2.0%, *P* = 0.012), and having casual partners (8.8, 6.8, 5.2, 4.4, and 4.6, *P* = 0.001) **2.** Condom use: At a total of four follow- up at 22 months, the proportion of workers who reported to use condom during intercourse with casual partners were 7.6, 23.5, 41.0, 25.8, 27.3%, *P* = 0.002. **3.** Sex behavior change: Low risk behavior (defined as none or one sexual partner) and high-risk behavior (defined as more than one sexual partner): With regard to the number of sexual partners in the last month, 61.7% reported low risk behavior at both the beginning and the end, 19.0% had changed from high to low, 12.2% continued high risk behavior, and 7.1% changed from low to high.					
	Mekonnen Y	2003	Cohort	Ethiopia	34 months	Combination of education and community intervention
**Sample size (before/after)**	1124 workers /921 workers					
**Intervention**	**1.** Health workers provided health education and HCT services. **2.** Health workers offered free medical care to factory workers and their families.					
**Outcome**	Declined in the proportion of workers reporting recent casual sex (from 17.5 to 3.5%, *P* < 0.001), sex with commercial sex worker (from 11.2 to 0.75%, *P* < 0.001), and genital discharge (from 2.1 to 0.6%, *P* = 0.004).					
	Sahlu T	2002	Cohort	Ethiopia	25 months	Combination of education and community intervention
**Sample size (before/after)**	757 workers /538 workers					
**Intervention**	Health workers provided HIV/AIDS health education, HCT services and free condoms in the factory.					
**Outcome**	**1.** Declined in the proportion of males reporting recent casual sex (from 12 to 6.1%, *P* = 0.03), sex with sex worker (from 3.5 to 1.0%, *P* = 0.07), genital discharge (from 2.1 to 1.5%, *P* > 0.05), and genital ulcer (from 0.4 to 1.0%, *P* > 0.05) in the last 25 months. **2.** Declined in the proportion of females reporting recent casual sex (from 2.2 to 0%, *P* = 0.03), genital discharge (from 12.9 to 8.9%, *P* > 0.05), and genital ulcer (from 3.6 to 2.1%, *P* > 0.05) in the last 25 months.					
	Machekano R	2000	Cohort	Zimbabwe	46 months	Combination of peer education and community intervention
**Sample size (before/after)**	3383 workers /NR					
**Intervention**	**1.** Providing HCT services, including individual risk assessment. **2.** Peer educators provided free condoms in the workplace, organized HIV/AIDS prevention drama, and arranged presentations. **3.** Peer educators led group discussions, distributed education materials, put up posters, and arranged video and slide shows.					
**Outcome**	**1.** Whether to give peer education or not has no statistical significance for individuals whether to accept HCT. (odds ratio (OR) = 1.05, 95%CI: 0.92–1.20, *P* = 0.484) **2.** Workers who received peer education were more willing to take their partners to HCT. (OR = 1.37, 95%CI: 1.04–1.79, *P* = 0.028) **3.** Workers with STDs were more likely to accept HCT (OR = 2.78, 95%CI: 2.25 to 3.43) and took their partners to HCT (OR = 3.67, 95%CI: 2.90 to 4.63). **4.** Workers who used to have ever paid for sex were more willing to go to HCT (OR = 1.27, 95%CI:1.09 to 1.49). **5.** Worker with multiple sex partners were more likely to go to HCT (OR = 1.31, 95%CI: 1.14 to 1.50) and preferred to take their partners to HCT (OR = 1.46, 95%CI: 1.11 to 1.92).					
	Weihs M	2014	Cohort	South African	2 weeks	Combination of lottery and community intervention
**Sample size (before/after)**	203 workers /NR					
**Intervention**	**1.** The first step of the experimental intervention was the announcement of the lottery incentive system (LIS). **2.** A leaflet was distributed to all workers approximately two weeks before workplace HCT services. **3.** Workers who participated in workplace HCT would receive free t-shirts and would be entered into a company lottery which afforded opportunities to win gift cards (a first prize of 2000 South African rand (ZAR), a second prize of 500 ZAR, and 10 extra 100 ZAR prizes).					
**Outcome**	Compared with the pre- and post-intervention, the uptake rate of HCT increased from 30 to 85%, *P* < 0.001.					
	Chamratrithirong A	2017	Cohort	Thailand	NR	Combination of policy and education intervention
**Sample size (before/after)**	Intervention group: 17 factories (NR/424 workers) Control group: 11 factories (NR/275 workers)					
**Intervention**	**1.** Policy intervention: To issue ASO certificates to factories, these factories must have non-discriminatory policies and confidentiality procedures for HIV-positive workers, support and care programs for HIV-infected workers, and have HIV/AIDS education programs for all workers, etc. **2.** Distribution of free condoms and installation of condom vending machines. **3.** Setting up HIV/AIDS exhibitions and handbooks.					
**Outcome**	This intervention method was significantly and positively related to HIV/AIDS knowledge (t = 2.834, *P* < 0.01), perceived condom accessibility (OR = 2.788 95%CI: 1.134 to 6.855, *P* < 0.05), and condom use with regular partners (OR = 1.247, 95%CI: 1.010, 1.540, *P* < 0.05).					
	Weihs M	2018	Cohort	South Africa	10 months	Combination of lottery and community intervention
**Sample size (before/after)**	Intervention group: 110 workers /101 workers Control group: 88 workers /84 workers					
**Intervention**	**1.** Firstly, educating all workers about HIV transmission, treatment, testing, and the importance of HCT; **2.** Second, setting up HCT service points and issuing brochures for the intervention factories; **3.** After 2 weeks, workers who participated in the workplace HCT could enter a lottery and had a chance to win money.					
**Outcome**	**1.** Lottery intervention reduced HIV stigma among factory workers. (22.2% in intervention group, 9.6% in control group, *P* < 0.05) **2.** HCT uptake in intervention group was higher than that in control group (53.6% in intervention group, 27.3% in control group, *P* < 0.001).					

Key: *NR* Not report; *RCT* Randomized control trial

The types and definitions of HIV intervention among factory workers in LMICs are summarized in Table 3. Further details on the risk of bias are reported in Additional file 3: Table S2. In selection bias, seven articles [18, 20, 32, 37–40] were rated “strong” because those study participants were factory workers, and more than 80% of the selected individuals agreed to participate. Six articles [19, 31, 33–36] were rated “medium” because only 60–79% of the selected individuals agreed to participate. In study design, two studies [31, 37] were randomized controlled trials (RCTs), so they were rated “strong”, and eleven studies [18–20, 32–36, 38–40] were cohort studies, so they were rated “medium”. In confounders, two of the thirteen studies [35, 40] were rated as “strong”, five as “medium” [18, 34, 36, 38, 39] and the remaining six [19, 20, 31–33, 37] as “weak”. For two RCTs [31, 37], the authors did not report whether confounding factors were balanced at baseline, so they were rated “weak”. In blinding methods, twelve [18, 19, 31–40] were rated “medium” or “weak” because the evaluator or participant knew the task of the study group. In data collection methods, the research data in most studies [18, 20, 32–40] were obtained from the survey and proved to be effective, so they were rated as “strong”, but there were two studies [19, 31] that did not evaluate the quality of the acquisition method (such as validity and reliability), so they were rated as “weak”. Finally, withdrawals and drop-outs were not related to the six studies [20, 33–35, 38, 40], as they did not include subsequent evaluations. Overall, five cohort studies were assessed as strong quality [18, 34, 35, 39, 40], four as moderate quality [20, 32, 36, 38], and two as weak quality [19, 33]. Two RCTs only show that RCT method was used, but did not describe how to control mixing and how to achieve blind method, so their final evaluation results were weak [31, 37].

**Table 3:** Categories of HIV interventions among factory workers

Intervention categories	Definition	Examples
Educational intervention	Health workers organize and implement interventions through expert lectures, group discussions and publicity materials	**1.** Health workers provide Cartoons, posters, radio programs, lectures and drama about HIV/AIDS. **2.** Health workers organize group discussions about condom use skills. **3.** Health workers manage and provide free condoms and contraceptives in the workplace.
Peer education	Peer educators intervene through peer communication	**1.** Peer educators provide free condoms in the workplace. **2.** Peer educators organize plays, speeches and discussions about HIV.
Community intervention	Intervention through active provision of HCT services and/or physical and mental health knowledge.	**1.** Individual risk assessment and blood test for HIV seroconversions. **2.** Reasonable diet, exercise, and injury prevention, such as influenza or workplace injury prevention.
Lottery intervention	Improvement of HCT uptake among factory workers through lottery drawing.	The first step was to publicize the lottery intervention system. The second step was to distribute the process manual 2 weeks before the intervention. The third step was to award free T-shirts to workers participating in HCT in the workplace, and to be able to participate in lottery activities to win prizes and gift cards.
Policy intervention	Encourage factory workers to acquire HIV/AIDS knowledge and reduce HIV discrimination by issuing certificates.	A policy intervention encouraged workers to learn HIV/AIDS knowledge by issuing AIDS-response Standard Organization (ASO) certificates.

### Efficacy of different intervention methods among factory workers

#### Educational intervention

Three studies focused on educational intervention [18, 37, 39]. Two studies indicated that educational intervention might improve condom use (condomless sex in the last 12 months decreased from 6.9% at baseline to 3.8% at month 12, *P* < 0.001; use of condom during sexual intercourse in the last 3 months increased from 41% at baseline to 70% at month 3, *P* < 0.05) [18, 39]. Two studies showed that educational intervention could improve HIV/AIDS knowledge [18, 37]. For example, workers who learned that antibiotics did not prevent HIV transmission increased from 46.9 to 56.3% (*P* = 0.03) and that mother-to-child could spread HIV increased from 82.6 to 93.4% (*P* < 0.05). One study showed that educational intervention could reduce the proportion of workers with extra-partners (from 16 to 5%, *χ*^2^ = 5.32, *P* = 0.021) [37]. One study showed that educational intervention could reduce the proportion of workers who used recreational drugs (from 2.6 to 0.7%, *P* < 0.01) or alcohol (from 17.0 to 6.3%, *P* < 0.01) before sex [18]. In addition, educational intervention changed HIV attitudes. For example, the proportion of workers who perceived that if they had HIV/AIDS they would not be able to live in society decreased from 46.6 to 30.6% (*P* < 0.05) [18].

#### Community intervention

One study conducted community intervention [32]. The study indicated that workers having high risk behaviors were more likely to take HCT, and proactive provision of HCT could increase the detection rate of HIV (relative risk [RR]: 1.87, 95% confidence interval [CI]: 1.01 to 3.61) and sexually transmitted diseases (STDs) (RR: 3.47, 95%CI: 2.51 to 4.89). Moreover, HIV seroconversion was higher among subjects who obtained their test results at the first follow-up visit compared to those who did not (19.5% vs. 16.7%, respectively, *P* = 0.01).

#### Combination of lottery intervention and community intervention

Two studies focused on lottery intervention combined with community intervention, both of which analyzed the changes in HCT uptake before and after the intervention [20, 33]. Moreover, these studies had demonstrated that lottery intervention could improve HCT uptake (from 30 to 85% (*P* < 0.001) [33] and from 27.3 to 53.6% (*P* < 0.001) [20]). In addition, lottery intervention could also reduce HIV public stigma. For example, the proportion of subjects who thought that foreigners/migrant workers/prostitutes were to blame for spreading HIV/AIDS decreased from 22.2 to 9.6% (*P* < 0.05) [20].

#### Combination of educational intervention and community intervention

Four studies conducted educational intervention combined with community intervention [34–36, 40]. Three studies demonstrated that educational intervention combined with community intervention reduced the proportion of workers with casual sex (from 12.0 to 6.1%, *P* = 0.03 [34]; from 17.5 to 3.5%, *P* < 0.001 [35]; from 8.8 to 4.6%, *P* < 0.01 [36]). Two studies showed a decrease in the proportion of workers having sex with sex workers [34, 35], but only one report [35] had statistically significant result (from 11.2 to 0.75%, *P* < 0.001 [35]). One study reported a decrease in the proportion of workers who had more than one sex partner. For example, the proportion of workers with two sexual partners decreased from 17 to 10% (*P* < 0.05) and with three or more sex partners decreased from 4.7 to 2.0% (*P* < 0.05) [36]. In addition, the combination of these two interventions increased condom use (from 7.6 to 27.3%, *P* = 0.002) [36], reduced premarital sex (10.9% in intervention group, 31.3% in control group, *P* < 0.001) [40], and improved HIV knowledge (*P* < 0.05) [40] and an increased awareness of the locations providing free health educational counselling (from 3.5 to 6.7%, *P* < 0.001) [40].

#### Combination of peer education and community intervention

Two studies focused on peer education combined with community intervention [19, 31]. One study indicated that peer education reduced incident HIV infection rate (1.51 vs. 2.52 per 100 persons-years, *P* < 0.05) [31]. Another study concluded that peer education rendered more workers to take their partners to HCT (odds ratio [OR] = 1.37, 95% CI: 1.04–1.79), but statistical significance was not found for individuals to take up HCT (OR = 1.05, 95% CI: 0.92–1.20) [19]. In addition, workers with STDs (OR = 2.78, 95%CI: 2.25–3.43), commercial sex (OR = 1.27, 95%CI:1.09–1.49) and multiple sex partners (OR = 1.31, 95%CI: 1.14–1.50) in the last 6 months were more likely to take up HCT [19].

#### Combination of policy intervention and educational intervention

One study conducted policy intervention combined with educational intervention [38]. This study indicated that combination of these intervention increased HIV/AIDS knowledge (t = 2.84, *P* = 0.005), perceived condom accessibility (OR = 2.80, 95% CI: 1.13–6.86, *P* < 0.05), and condom use with regular partners (OR = 1.25, 95% CI: 1.01–1.54, *P* < 0.05) at the last sex.

## Discussion

Our systematic review identified five types of interventions that addressed low HIV/AIDS knowledge, high risk behaviors of HIV infections, high HIV stigma and low HCT uptake among factory workers in LMICs, namely educational intervention, peer education, community intervention, lottery intervention and policy intervention. Educational intervention and policy intervention had a significant effect on improving workers’ knowledge of HIV/AIDS and reducing HIV infection risk behaviors. Community intervention, peer education and lottery intervention were effective in reducing HIV public stigma and increasing HCT uptake.

In this review, most of intervention methods from the included studies were in combination. Among them, community intervention was combined with a variety of interventional methods. This intervention focused primarily on the socio-demographic characteristics and HIV serological status of the workers who underwent HCT [42]. Relevant studies showed that most people who attended HCT were at high risk of HIV infection [43]. Individuals infected with HIV were tested earlier than those who were not, and those who were in the early stages of HIV infection were tested earlier than those who were in the late stages [43]. This review also showed that community intervention demonstrated that the willingness of high-risk workers was likely to attend HCT when offered this opportunity. A recent cohort study in South Africa, demonstrated a similar outcome with a community intervention [44]. These findings suggested that offering HCT services to study subjects could potentially detect HIV infected individuals on a timely basis. In addition, the benefit of HCT could also be improved by active screening for STDs [45].

Although the efficacy of single community intervention was limited, it was significantly better when combined with different interventions. For example, community intervention combined with lottery intervention could improve HCT uptake and reduce HIV public stigma. This effect mainly came from lottery intervention. Lottery intervention encouraged workers to attend HCT by giving away free T-shirts, winning gifts and money and thus provided workers with an opportunity with a forum for open discussion [17, 46]. Social support and encouragement made these workers more willing to improve their HIV knowledge [47]. Social support could promote individual self-esteem and was a key factor in enabling the intervention to proceed smoothly and promote effectiveness [48, 49]. Research has shown that increasing social support and personal self-esteem could effectively reduce HIV stigma [49], which are essential for the UNAIDS to implement the three 90% [50] targets by 2030 (Three 90% prevention and treatment strategies of HIV: 90% of PLWH will know their infection status through testing, 90% of PLWH who have been diagnosed will have received antiviral therapy, and 90% of PLWH who have received antiviral therapy will be able to successfully suppress HIV).

Educational intervention was the most widely applied in HIV/AIDS interventions, and achieved a variety of effective results. Educational intervention had a significant effect on improving HIV/AIDS knowledge, changing HIV attitudes and reducing the proportion of workers with extra-partners, and could also change premarital and paid sexual behavior when combined with community intervention. A recent systematic review showed that educational intervention combined with community intervention could also change the public stigma of HIV [51]. In the future, more attention should be paid to HIV education combined with community intervention [52]. The combination effect of educational intervention and community intervention is better than a single intervention [53, 54]. Highly effective interventions, such as those that have an educational component, are imperative to undertake, but the cost of these designs - especially in LMIC - often limit their implementation even though they demonstrate high uptake of HCT and lower risky behaviors [54, 55]. Cost-effectiveness analysis provides information to help us balance the cost and efficacy when we implement educational intervention [54], so more research is needed to analyze the cost-effectiveness of an educational intervention.

Peer education combined with community intervention was also an effective intervention method, which could result in promoting workers to bring their partners to HCT and encouraging workers with high risk sexual behaviors to carry out HCT. According to recent research, peer education and community intervention not only improved HIV/AIDS knowledge, reduced HIV risk behaviors and incidence, but also increased HCT uptake in high-risk groups [56, 57]. Other relevant research showed that peer education could improve HIV knowledge, change HIV attitudes and reduce risky sexual behavior among adolescents, especially within peer groups [58, 59]. Peers are more likely to influence the behavior of fellow group members because they gain a level of trust that allows them to have more open discussions about sensitive topics [58, 59]. Therefore, in order to analyze whether peer education can achieve this same effect among factory workers, more research is needed.

This review summarized two special intervention methods, lottery intervention and policy intervention. These two interventions were based on incentives, and their purpose was to encourage workers to access HCT services. Policy intervention encourages workers to learn HIV/AIDS knowledge by issuing AIDS-response Standard Organization (ASO) certificates [60], while a lottery intervention encouraged workers to attend HCT by giving away free T-shirts, winning gifts and money and provided workers with an opportunity with a forum for open discussion [33]. Although this review summarized some benefits of these two interventions, only three relevant articles had been included. Further investigation and research are needed to explain their specific efficacy and causes.

In the 13 articles included, we did not find any research on interventions using multimedia or smart devices among factory workers. Reviews have shown that short message service (SMS) and interventions using mobile phone software can significantly improve HIV testing in high-risk groups [61, 62]. In addition, the use of multimedia or intelligent devices can effectively improve the uptake of intervention measures [61]. Therefore, for factory workers in LMICs - a group at high risk of HIV infection - It’s urgent to examine the efficacy of multimedia and smart devices-based interventions.

This study has several limitations. Firstly, it is impossible for us to judge which intervention brought about effect if multiple interventions were involved. With this in mind, more effort should be focused on single intervention or a combination of multiple interventions to compare the efficacy of various interventions in HIV high-risk groups. Secondly, articles included in our study did not analyze the cost-effectiveness and uptake of various methods, which is a key factor to the implementation of an intervention. Thirdly, since we only retrieved articles from four databases (PubMed, PsycINFO, Scopus and EMBASE), we might have missed some relevant articles from other sources (e.g., Google Scholar, EBSCOhost and WEB of Science). Finally, even if the indicators are the same, the methods of index measurement may differ such as the setting of HIV/AIDS knowledge and condom use measurement methods, which could contribute to detection bias.

## Conclusions

This review indicated that various HIV interventions were efficacious in improving HIV knowledge and reducing HIV-related high-risk behaviors among factory workers in LMICs. The effectiveness of single intervention is limited and the combination of multiple interventions could achieve better outcomes. The efficacy of multimedia and smart devices-based interventions is warranted to be examined in the future.

## Supplementary information

**Additional file 1:** Efficacy of HIV interventions among factory workers in low- and middle-income countries protocol of a systematic review.

**Additional file 2:** PubMed, EMBASE, Scopus and PsycINFO search strategy.

**Additional file 3:** Quality assessment of 13 studies of HIV interventions among factory workers in LMICs.

## Data Availability

All data are provided in the tables, figure, and Additional files presented in the text. The other materials can be made available upon request.

## Abbreviations

HIV: Human immunodeficiency virus
LMICs: Low- and middle-income countries
HICs: High-Income Countries
AIDS: Acquired immune deficiency syndrome
UNAIDS: United Nations Program on HIV/AIDS
MSM: Men who have sex with men
HCT: HIV counselling and testing
RCT: Randomized controlled trial
PRISMA: Preferred Reporting Items for Systematic Reviews and Meta-Analyses
PICOS: Population, intervention, comparison, outcomes, and study design
RR: Relative risk
CI: Confidence interval
STDs: Sexually transmitted diseases
OR: Odds ratio
SMS: Short message service
ASO: AIDS-response Standard Organization
EPHPP: Effective Public Health Practice Project
