# Supplementary material for: Efficacy of HIV interventions among factory workers in low- and middle-income countries: a systematic review
Source: BMC Public Health. 2020 Aug 28;20:1310. doi: 10.1186/s12889-020-09333-w (PMC7455896; doi:10.1186/s12889-020-09333-w)
Supplement: Supplementary file 1 — Additional file 1: Efficacy of HIV interventions among factory workers in low- and middle-income countries protocol of a systematic review. [file 12889_2020_9333_MOESM1_ESM.docx]

Efficacy of HIV interventions among factory workers in low- and middle-income countries: protocol of a systematic review

**Background**

Factory workers in low- and middle-income countries (LMICs) are vulnerable to HIV transmission. According to a recent study in 2019, Lesotho national textile factory workers had a higher HIV infection rate than all adults in the country (42.7% vs 25%, *P* < 0.05) [1]. Various factors influence the susceptibility of factory workers to HIV, including low level of education, high incidence of high-risk sexual behaviors and low HIV testing rate, etc. Some interventions, including an educational intervention, a peer education, and financial incentives, can produce beneficial outcomes in this population. However, to our knowledge, there are no published review about summarizing and comparing the efficacy of these interventions. Thus, we will perform a systematic review to evaluate the efficacy of various interventions among factory workers in LMICs.

**Study aims**

This study aims to review published literature on the efficacy of various HIV interventions in reducing stigma, risk behaviors and HIV transmission among factory workers.

**Study design**

A systematic review among factory workers in low- and middle-income countries.

**Methods**

This systematic review will be followed the guidelines set forth in the 2010 ‘Preferred Reporting Items for Systematic Reviews and Meta-Analyses (PRISMA)’ [2].

**Search strategy**

The Mesh terms “workplace/industry/acquired immunodeficiency syndrome” and the key word “worker” will be combined using the Boolean operator and with the following key words: (enterprise, firm, company, workshop, floor shop, machine shop, mill, factory, manufactory) and (worker, workman, workingman, employee). Key words in parentheses will be connected to operators. The search strategy will be implemented in the databases PubMed, PsycINFO, Scopus and EMBASE using a date range of January 1, 1990 through December 31, 2018. Grey literature online (e.g., AIDS Conference, International AIDS Society Conference) also will be tried to searched.

**Search strategy for PubMed**

#1. Search ((((((((((((((enterprise*[Title/Abstract]) OR firm*[Title/Abstract]) OR compan*[Title/Abstract]) OR workshop*[Title/Abstract]) OR flow shop*[Title/Abstract]) OR machine shop*[Title/Abstract]) OR mill*[Title/Abstract]) OR factory[Title/Abstract]) OR factories[Title/Abstract]) OR manufactor*[Title/Abstract]) OR manufacturer*[Title/Abstract]) OR work place*[Title/Abstract])) OR "Workplace"[Mesh]) OR "Industry"[Mesh]

#2. Search ((((((worker*[Title/Abstract]) OR workman [Title/Abstract]) OR workmen [Title/Abstract]) OR laborer*[Title/Abstract]) OR workingman [Title/Abstract]) OR workingmen [Title/Abstract]) OR employee*[Title/Abstract]

#3. #1 AND #2

#4. Search ("Acquired Immunodeficiency Syndrome"[Mesh]) OR ((HIV[Title/Abstract]) OR AIDS[Title/Abstract])

#5. #3 AND #4

#6. Search (sex worker [Title/Abstract]) OR sex workers [Title/Abstract]

#7. #5 NOT #6 Filters: Publication date from 1990/01/01 to 2018/12/31

Search strategy for EMBASE

(((enterprise* OR firm* OR compan* OR workshop* OR (('flow'/exp OR flow) AND shop*) OR (('machine'/exp OR machine) AND shop*) OR mill* OR 'factory'/exp OR factory OR factories OR manufactor* OR manufacturer* OR (('work'/exp OR work) AND place*) OR workplace* OR industr*:ab,kw,ti) AND (worker* OR workman OR workmen OR laborer* OR workingman OR workingmen OR employee*:ab,kw,ti)) AND ('human immunodeficiency virus'/exp OR 'acquired immune deficiency syndrome'/exp OR (aids OR hiv:ab,kw,ti))) NOT ('sex worker' OR 'sex workers':ab,kw,ti) AND [1-1-1990]/sd NOT [31-12-2018]/sd

The two authors (DC and GL) will search for relevant articles independently. Titles, abstracts, full texts and reference lists of all identified reports will be reviewed in duplicate by the two authors, and extracted articles will be double-checked. Disagreements will be resolved by discussion among the three authors (DC, GL and HZ). Reference lists from related main studies and review articles were also checked for additional relevant reports.

**Eligibility Criteria**

The inclusion criteria will be: (1) The scope formulate using the population, intervention, comparison, outcomes, and study design (PICOS) format (see Table1); (2) Studies conduct in LMICs according to the World Bank [3]; (3) Studies report specific intervention time span; (4) Articles were written in English.

Studies will be excluded based on the following criteria: (1) participants were not factory workers; (2) no intervention; (3) article published before 1990; (4) Studies were observational, and did not describe an intervention’s efficacy on reducing HIV infection, changing HIV risk behaviors and attitudes, and decreasing HIV stigma (e.g., a cross-sectional study); (5) systematic review, literature review, case series; (6) article published in languages other than English.

**Data Extraction**

The following data will be extracted from publications: year of publication, first author, country in which the study took place, study design, sample size, length of follow-up, intervention method, and intervention outcomes.

**Quality Assessment of Included Studies**

In order to assess the quality of the included articles, we will use the Quality Assessment Tool for Quantitative studies from the Effective Public Health Practice Project (EPHPP) [4, 5]. This tool has been widely used in literature to evaluate randomized control trials of HIV research [6, 7], and was recommended by the Cochrane Library in the area of Health Promotion and Public Health [8, 9]. Quality assessment include 6 components: selection bias, study design, confounders, blinding methods, data collection method, and withdrawals and drop-outs. The scores of each component will be based on the documents [4, 5]. A study will be received a "strong" overall rating when none of the individual components has been rated as “weak”. If a study cannot get more than one “weak” score on any single component, it will be received a "medium" overall rating. A study of at least two “weak” ratings for individual components will be given a "weak" overall rating.

**Reference**

1. Mabathoana RS, Wyk CV, Adefuye AO: Factors influencing HIV risk-taking behaviours amongst textile factory workers living with HIV in Lesotho. The Pan African Medical Journal 2019, 33:166.

2. Moher D, Liberati A, Tetzlaff J, Altman DG, Group P: Preferred reporting items for systematic reviews and meta-analyses: the PRISMA statement. International Journal of Surgery 2010, 8(5):336-341.

3. World Bank Group [US]: low- and middle-income countries in 2017. In: 2017. <https://data.worldbank.org.cn/income-level/low-and-middle-income?view=chart>. Accessed May 10, 2019.

4. Project EPHP: Quality assessment tool for quantitative studies. In: 1998. <https://merst.ca/ephpp/>. Accessed May 10, 2019.

5. Thomas BH, Ciliska D, Dobbins M, Micucci S: A process for systematically reviewing the literature: providing the research evidence for public health nursing interventions. Worldviews on Evidence-Based Nursing 2004, 1(3):176-184.

6. Genberg BL, Shangani S, Sabatino K, Rachlis B, Wachira J, Braitstein P et al: Improving Engagement in the HIV Care Cascade: A Systematic Review of Interventions Involving People Living with HIV/AIDS as Peers. AIDS and Behavior 2016, 20(10):2452-2463.

7. Shangani S, Escudero D, Kirwa K, Harrison A, Marshall B, Operario D: Effectiveness of peer-led interventions to increase HIV testing among men who have sex with men: a systematic review and meta-analysis. AIDS Care 2017, 29(8):1003-1013.

8. Jackson N, Waters E: Criteria for the systematic review of health promotion and public health interventions. Health Promotion International 2005, 20(4):367-374.

9. Armstrong R, Waters E, Jackson N: Guidelines for systematic reviews of health promotion and public health interventions. Version 2 Australia: Melbourne University. In: 2007. <http://ph.cochrane.org/sites/ph.cochrane.org/files/public/uploads/Guidelines%20HP_PH%20reviews.pdf>. Accessed May 10, 2019.
