# Supplementary material for: Efficacy of HIV interventions among factory workers in low- and middle-income countries: a systematic review
Source: BMC Public Health. 2020 Aug 28;20:1310. doi: 10.1186/s12889-020-09333-w (PMC7455896; doi:10.1186/s12889-020-09333-w)
Supplement: Supplementary file 2 — Additional file 2: PubMed, EMBASE, Scopus and PsycINFO search strategy. [file 12889_2020_9333_MOESM2_ESM.docx]

**Table S1：**PubMed, EMBASE, Scopus and PsycINFO search strategy

| **Search strategy for PubMed**  #1. Search ((((((((((((((enterprise*[Title/Abstract]) OR firm*[Title/Abstract]) OR compan*[Title/Abstract]) OR workshop*[Title/Abstract]) OR flow shop*[Title/Abstract]) OR machine shop*[Title/Abstract]) OR mill*[Title/Abstract]) OR factory[Title/Abstract]) OR factories[Title/Abstract]) OR manufactor*[Title/Abstract]) OR manufacturer*[Title/Abstract]) OR work place*[Title/Abstract])) OR "Workplace"[Mesh]) OR "Industry"[Mesh]  **574762 records**  #2. Search ((((((worker*[Title/Abstract]) OR workman [Title/Abstract]) OR workmen [Title/Abstract]) OR laborer*[Title/Abstract]) OR workingman [Title/Abstract]) OR workingmen [Title/Abstract]) OR employee*[Title/Abstract]  **197677 records**  #3. #1 AND #2  **39753 records**  #4. Search ("Acquired Immunodeficiency Syndrome"[Mesh]) OR ((HIV[Title/Abstract]) OR AIDS[Title/Abstract])  **376460 records**  #5. #3 AND #4  **908 records**  #6. Search (sex worker [Title/Abstract]) OR sex workers [Title/Abstract]  **4963 records**  #7. #5 NOT #6 Filters: Publication date from 1990/01/01 to 2018/12/31  **732 records** |
| --- |
| **Search strategy for EMBASE**  (((enterprise* OR firm* OR compan* OR workshop* OR (('flow'/exp OR flow) AND shop*) OR (('machine'/exp OR machine) AND shop*) OR mill* OR 'factory'/exp OR factory OR factories OR manufactor* OR manufacturer* OR (('work'/exp OR work) AND place*) OR workplace* OR industr*:ab,kw,ti) AND (worker* OR workman OR workmen OR laborer* OR workingman OR workingmen OR employee*:ab,kw,ti)) AND ('human immunodeficiency virus'/exp OR 'acquired immune deficiency syndrome'/exp OR (aids OR hiv:ab,kw,ti))) NOT ('sex worker' OR 'sex workers':ab,kw,ti) AND [1-1-1990]/sd NOT [31-12-2018]/sd  **1612 records** |
| **Search strategy for Scopus**  #1. (TITLE-ABS-KEY (enterprise*) OR TITLE-ABS-KEY (firm*) OR TITLE-ABS-KEY (workshop*) OR TITLE-ABS-KEY (factory) OR TITLE-ABS-KEY (factories) OR TITLE-ABS-KEY (workplace) OR TITLE-ABS-KEY (mill*) OR TITLE-ABS-KEY (industr*))  **4001270** **records**  #2. (TITLE-ABS-KEY (worker*) OR TITLE-ABS-KEY (workm*n) OR TITLE-ABS-KEY (workingm*n) OR TITLE-ABS-KEY (employee*))  **605780** **records**  #3. (TITLE-ABS-KEY (human AND immunodeficiency AND virus) OR TITLE-ABS-KEY (acquired AND immune AND deficiency AND syndrome) OR TITLE-ABS-KEY (hiv) OR TITLE-ABS-KEY (aids))  **616988** **records**  #4. #1 AND #2 AND #3，date 1990-2018  **2277** **records** |
| **Search strategy for** **PsycINFO**  #1. AB enterprise* OR AB firm* OR AB workshop* OR AB flow shop* OR AB factory OR AB factories OR AB Workplace  **94221** **records**  #2. AB worker* OR AB workman OR AB workingman OR AB employee* OR AB laborer*  **134179** **records**  #3. AB Acquired Immunodeficiency Syndrome OR AB HIV OR AB AIDS OR AB acquired immune deficiency syndrome  **62082** **records**  #4. #1 AND #2 AND #3，1990-2018  **235** **records** |
