# Supplementary material for: Efficacy of HIV interventions among factory workers in low- and middle-income countries: a systematic review
Source: BMC Public Health. 2020 Aug 28;20:1310. doi: 10.1186/s12889-020-09333-w (PMC7455896; doi:10.1186/s12889-020-09333-w)
Supplement: Supplementary file 3 — Additional file 3: Quality assessment of 13 studies of HIV interventions among factory workers in LMICs. [file 12889_2020_9333_MOESM3_ESM.docx]

**Table S2** Quality assessment of 13 studies of HIV interventions among factory workers in LMICs

| Primary author | Publication year | Selection Bias | Study design | Confounders | Blinding | Data collection method | Withdrawals and drop-outs | Overall quality rating |
| --- | --- | --- | --- | --- | --- | --- | --- | --- |
| Kuchaisit C | 1996 | 1 | 1(RCT) | 3 | 3 | 1 | 1 | 3 |
| Sakondhavat C | 1998 | 1 | 2(Cohort) | 2 | 2 | 1 | 1 | 1 |
| Machekano R | 1998 | 1 | 2(Cohort) | 3 | 2 | 1 | 1 | 2 |
| Bassett M | 1998 | 2 | 1(RCT) | 3 | 3 | 3 | 2 | 3 |
| Qian X | 2007 | 1 | 2(Cohort) | 2 | 2 | 1 | 2 | 1 |
| Zhu C | 2014 | 1 | 2(Cohort) | 1 | 2 | 1 | N/A | 1 |
| Ng'weshemi J | 1996 | 2 | 2(Cohort) | 2 | 2 | 1 | 3 | 2 |
| Mekonnen Y | 2003 | 2 | 2(Cohort) | 1 | 2 | 1 | N/A | 1 |
| Sahlu T | 2002 | 2 | 2(Cohort) | 2 | 2 | 1 | N/A | 1 |
| Machekano R | 2000 | 2 | 2(Cohort) | 3 | 3 | 3 | 2 | 3 |
| Weihs M | 2014 | 2 | 2(Cohort) | 3 | 3 | 1 | N/A | 3 |
| Chamratrithirong A | 2017 | 1 | 2(Cohort) | 2 | 3 | 1 | N/A | 2 |
| Weihs M | 2018 | 1 | 2(Cohort) | 3 | 1 | 1 | N/A | 2 |

Key: 1=strong, 2=moderate, 3=weak, N/A=not applicable, RCT=randomized controlled trial
